# Identification of *VEGFB* associated with NKT cells in diabetic foot ulcers: Single-cell analysis and machine learning

**DOI:** 10.1097/MD.0000000000047574

**Published:** 2026-02-13

**Authors:** Weilun Wang, Yao He, Tao You, Meihong Lei, Jianming Chen

**Affiliations:** a Department of Endocrinology, The 909th Hospital, School of Medicine, Xiamen University, Zhangzhou, Fujian, China.

**Keywords:** diabetes foot ulcers, machine learning, NKT cell, single cell analysis

## Abstract

The management options for diabetic foot are restricted, and the outlook is unfavorable. Immune cells have been implicated in diabetic foot ulcer (DFU), but the exact role of natural killer T (NKT) cells in DFU remains unclear. *Vascular endothelial growth factor B (VEGFB*), a member of the *VEGF* family, is distinguished by its potential roles in metabolic regulation and immune modulation, yet its connection to NKT cells in DFU is unexplored. This study was to identify specific genes associated with NKT cells in DFU and to ascertain potential targets. We analyzed single-cell ribonucleic acid sequencing and bulk transcriptome data from DFU datasets. Differential expression analysis identified genes associated with NKT cells in DFU. Machine learning algorithms were applied to pinpoint the most significant genes from these candidates. The functional characteristics of the identified key gene were further investigated through gene set enrichment analysis and immune infiltration analysis. Single-cell analysis revealed 390 NKT cell-related genes, and differential analysis identified 728 differentially expressed genes. Cross-referencing yielded 37 NKT cell-related differentially expressed genes. Machine learning consistently identified *VEGFB* as a key biomarker. Functional analysis linked *VEGFB* to cell adhesion, vasculature development, and angiogenesis pathways. *VEGFB* was significantly overexpressed in DFU samples compared to controls. Our study identifies *VEGFB* as a valuable biomarker associated with NKT cells in DFU. The overexpression of *VEGFB* suggests its involvement in DFU pathogenesis, potentially bridging immune regulation and vascular pathways. This finding enhances the understanding of NKT cell mechanisms in DFU and positions *VEGFB* as a potential target for future diagnostic and therapeutic strategies aimed at immunomodulation.

## 1. Introduction

Over the past few years, diabetes mellitus (DM) has slowly become an issue of global public health.^[1]^ DM is associated with several complications, in which diabetic foot ulcers (DFUs) represent a severe and prevalent condition.^[2]^ DFUs are a devastating complication and the leading cause of nontraumatic lower extremity amputations worldwide.^[3]^ It is estimated that approximately a quarter of diabetic individuals will develop a foot ulcer during their lifetime, and among these patients, the cumulative incidence of lower extremity amputation can reach 20%, which is associated with a dramatically increased mortality rate, up to 50% within 1-year postamputation.^[4]^ In DFU treatment, wound debridement, wound unloading, blood glucose regulation, and infection control are the most common techniques. Despite this, the therapeutic effect remains largely unsatisfactory.^[5,6]^ A deeper understanding of the underlying pathophysiological mechanisms driving the development and progression of DFUs is crucial for the formulation of novel therapeutic interventions.

The compromised healing of foot wounds in patients with DFU often leads to chronic wounds.^[6,7]^ The tissue repair process involves diverse cellular constituents, including endothelial cells, fibroblasts, keratinocytes, and immune cells. However, the precise contributions of specific immune cell subsets remain obscure. Notably, Natural Killer T (NKT) cells are a unique subset of T lymphocytes that bridge innate and adaptive immunity. They express an invariant T-cell receptor that recognizes lipid antigens presented by the Major histocompatibility complex-I-like molecule CD1d, rather than peptide antigens presented by conventional major histocompatibility complex molecules.^[8]^ Upon activation, NKT cells can rapidly produce a plethora of cytokines and exhibit direct cytotoxic activity, thereby potently modulating the immune microenvironment.^[9]^ Given their documented roles in regulating angiogenesis, inflammation, and fibrosis in various pathological contexts, we hypothesize that NKT cells may play a pivotal yet unexplored role in the impaired wound healing microenvironment of DFU.

Within the complex cellular milieu of DFU, growth factors are critical for healing. Among the *vascular endothelial growth factor (VEGF*) family, which includes *VEGF-A*, *VEGF*-B, *VEGF-C*, *VEGF-D*, and PIGF, *VEGF-A* has been extensively studied for its potent angiogenic effects. In contrast, *VEGF-B*, which primarily binds to *VEGFR-1*, has distinct functions that extend beyond angiogenesis, including potential roles in metabolic regulation and immune modulation, setting it apart from other *VEGF* family members.^[10,11]^ The specific involvement of *VEGF-B* in the context of DFU, particularly its potential link with NKT cells, remains largely unknown and represents a significant knowledge gap.

Hence, grounded upon the scrutiny of high-dimensional transcriptomic profiling and genomic data, this investigation employs machine learning methodologies to elucidate the mechanistic intricacies underlying NKT cell involvement in DFU. This endeavor aims to furnish an analytical and empirical foundation conducive to the amelioration of DFU.

## 2. Methods

### 2.1. DFU‐related datasets

**Figure 1.**
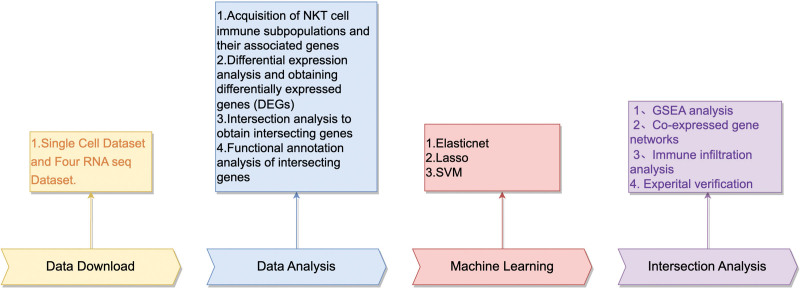
Flow chart of this study. DEG = differentially expressed genes, GSEA = gene set enrichment analysis, NKT = natural killer T, RNA = ribonucleic acid, SVM = support vector machine.

The research workflow is depicted in Figure 1. We gathered single-cell sequencing datasets related to DFU from the gene expression omnibus database: GSE165816. Additionally, we obtained 4 distinct transcriptome datasets: GSE68183, GSE80178,GSE134431, and GSE147890. Additionally, the “Combat” algorithm from the R package “SVA” was utilized to mitigate batch effects among the datasets, enabling the integration of the 4 distinct ribonucleic acid sequencing datasets into a unified dataset.

### 2.2. Single‐cell analysis

For cell clustering, we utilized the Seurat package to conduct t-SNE analysis and principal component analysis (PCA). Data filtration was used to ensure the integrity of our analysis, removing cells with mitochondrial gene counts below 200 or exceeding 2500, or comprising more than 5% of the total. Subsequently, after standardizing the data, we applied the “VST” method to identify 2000 highly variable genes. PCA was then conducted to identify crucial PCs, with 25 PCs chosen for the following dimensionality reduction using t-SNE. Finally, the cells were grouped into 19 distinct clusters with a resolution of 1.5.

The clustering resolution (resolution = 1.5) was determined by systematically testing a range of values (0.1 to 2.0 in 0.1 increments). We used the clustree package to visualize cluster stability across resolutions and calculated cluster purity metrics at each resolution using the ROGUE method. We ultimately selected 1.5 because, at this resolution, the cluster structure was clear and stable while enabling the identification of biologically relevant subpopulations with significantly differentially expressed genes. The number of principal components (PC = 25) was chosen based on the PCA ElbowPlot (scree plot), which showed an inflection point after the first 25 PCs, indicating that these components capture the most significant biological variation in the data.

### 2.3. Identification and functional annotation of differentially regulated genes

In analyzing DFU, we utilized the “limma” package to identify differentially expressed genes (DEGs), employing a threshold of |log2FC| > 1 and *P* < .05. We then created heatmaps and volcano plots for the DEGs using the R packages “Pheatmap” and “ggplot2.” Subsequently, we compared these DEGs with specific genes marking different macrophage subpopulations in the single-cell dataset. Following this, we annotated the intersecting genes for functionality. Finally, we conducted gene ontology (GO) and Kyoto Encyclopedia of Genes and Genomes (KEGG) pathway enrichment analyses to identify significant pathways with a *P*-value threshold < 0.05.

### 2.4. Identification of potential biomarkers for DFU based on 3 machine algorithms

We utilized 3 machine learning algorithms to discern potential biomarkers linked to DFU. These included Elasticnet, Lasso, and support vector machine (SVM). They were used to filter DFU features. We evaluated predictive performance via 10-fold cross-validation, focusing on genes with relative importance >1.5. Subsequently, we investigated these genes’ roles in disease progression and treatment. Additionally, we assessed biomarker diagnostic accuracy and analyzed gene expression among patients with DFU. Using independent *t*-tests, we compared biomarker expression in DFU patient and healthy individual samples, with significance established at *P* < .05. Diagnostic utility was evaluated using receiver operating characteristic (ROC) analyses.

To effectively control the risk of model overfitting, in addition to 10-fold cross-validation, we implemented the following measures: using the L1 and L2 regularization terms built into the Elastic Net and Lasso algorithms to penalize excessively large coefficients and simplify the model; performing feature selection via algorithm-internal feature importance assessments (e.g., Random Forest and Elastic Net) to retain genes with importance scores >1.5, thereby removing redundant features; and strictly evaluating model performance on independent test sets or on validation folds within cross-validation, rather than on the training data.

### 2.5. GSEA of biomarkers related to DFU

Based on DFU-associated biomarker expression levels, we categorized samples into groups based on their expression levels. Through gene set enrichment analysis (GSEA) and KEGG, we analyzed pathway enrichment in these groups, identifying significant gene sets with a nominal *P*-value < .05 and false discovery rate *q*-value < .25. GeneMANIA was utilized to explore genes co-expressed with biomarkers and to build gene networks.

### 2.6. Immune infiltration analysis

We used the CIBERSORT deconvolution method to assess the relative abundance of 22 immune cell types in tissue samples. This method quantifies the proportions of these cell types in the DFU cohort using linear support vector regression. We utilized Wilcoxon testing to evaluate variations in immune cell levels between samples from DFU patients and those from controls.

## 3. Results

### 3.1. Identification of marker genes for NKT cells related to DFU

**Figure 2.**
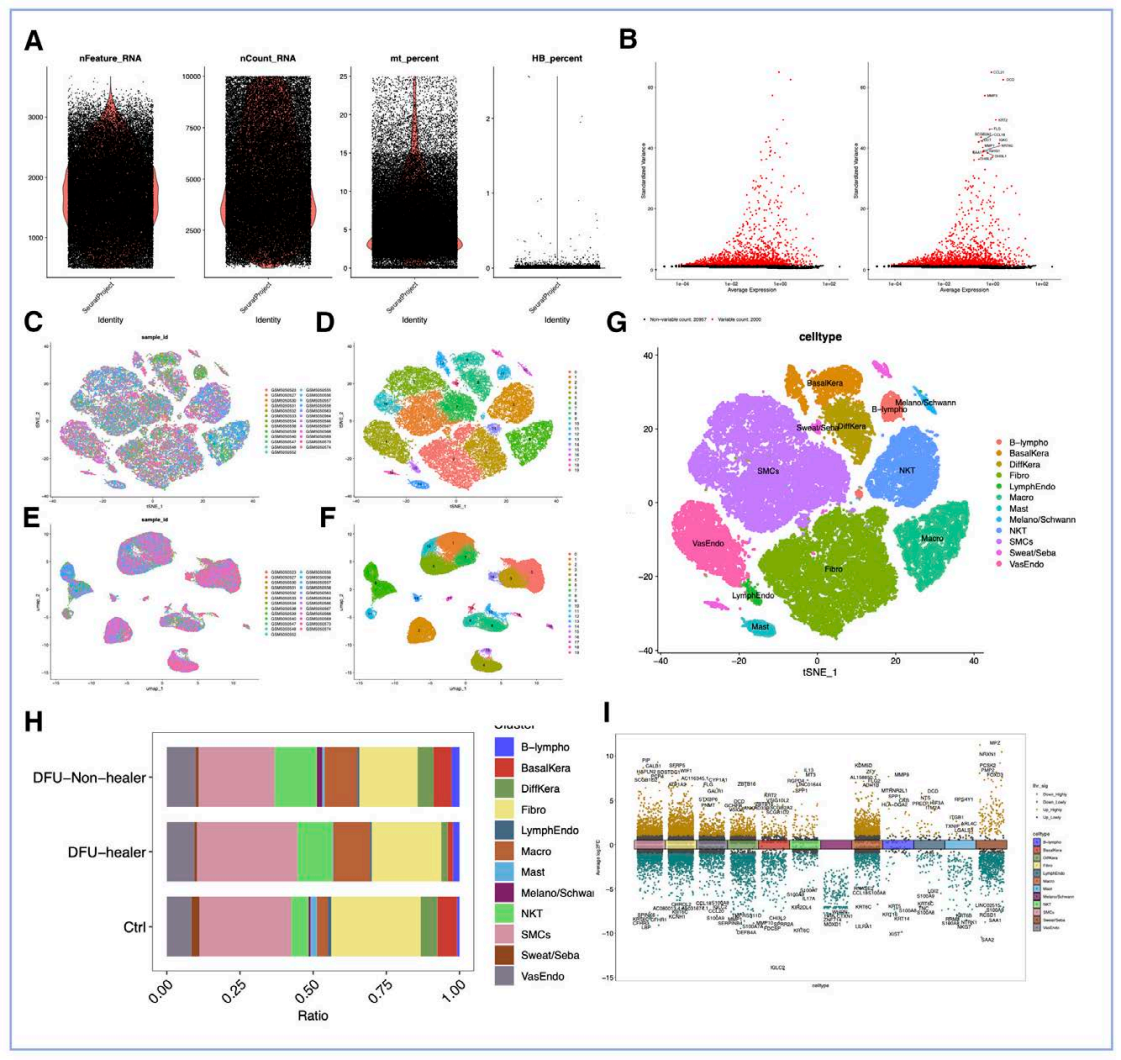
Identification of marker genes for the NKT subgroup. (A) Assessment of quality standards for single-cell data. (B) The depiction of 2000 highly variable genes by a set of red dots. (C–F) Classification of single-cell samples with annotations. (G) Annotation of single-cell subsets. (H) Comparison of immune cell infiltration between normal and DFU samples. (I) Evaluation of the enrichment of cytokines in various immune cell subsets. DFU = diabetic foot ulcer, NKT = natural killer T.

We conducted an intricate investigation employing single-cell analysis. In the initial stage, stringent quality control measures were implemented, whereby cells falling beyond the predefined standards were judiciously excluded (Fig. 2A). Following this crucial step, during the subsequent phase of data normalization, we meticulously handpicked a subset consisting of the most discerning and distinctive genes, totaling 2000 in number. Employing the method of PCA, we proficiently reduced the dimensionality of the dataset, paving the way for insightful interpretation (Fig. 2C).

By means of meticulous gene annotation, we astutely identified and delineated an impressive spectrum of 12 distinct cellular subtypes (Fig. 2C–G). At long last, in order to delve deeper into the intricate realm of NKT cell subgroups, we diligently targeted a select group of 390 genes for comprehensive analysis and exhaustive characterization (Fig. 2I).

### 3.2. Identification and functional annotation analysis of DEGs between normal and DFU samples

**Figure 3.**
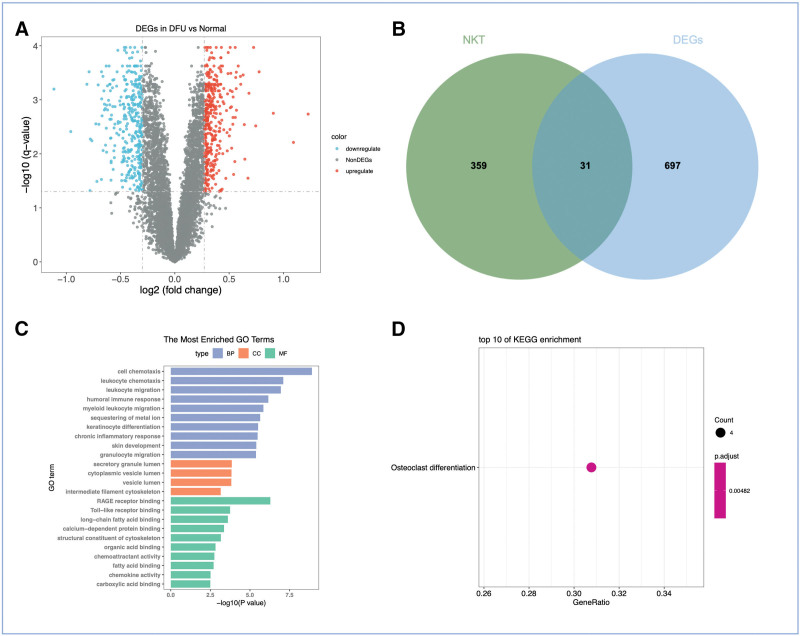
Identification of differentially expressed genes (DEG) between normal and DFU samples. (A) Volcano plot displaying all significant DEGs (red: upregulated in DFU; blue: downregulated; thresholds: |log2FC| > 1 and *P* < .05). (B) Venn diagram illustrating the overlap between the total DEGs and the NKT cell-related genes, yielding 31 intersecting genes. (C) Bar plot of gene ontology (GO) biological process terms significantly enriched in the 31 intersecting genes. (D) Bar plot of Kyoto Encyclopedia of Genes and Genomes (KEGG) pathways significantly enriched in the 31 intersecting genes. DEG = differentially expressed genes, DFU = diabetic foot ulcer, NKT = natural killer T.

We harmoniously amalgamated and meticulously normalized 4 publicly available gene expression omnibus datasets of substantial magnitude. Through rigorous analysis, we successfully discerned 743 DEGs, as convincingly illustrated in Figure 3A. Building upon this foundation, we embarked upon a comprehensive cross-analysis encompassing the DEGs and the specifically labeled genes pertaining to NKT cell subtypes. This intricate examination yielded remarkable findings, unveiling a total of 31 genes exhibiting significant correlations (Fig. 3B).

Delving deeper into the functional significance of these genes, GO analysis shed light on their pivotal roles within diverse cellular processes (Fig. 3C). Specifically, within the BP category, these genes exhibited pronounced enrichment in crucial activities such as cell chemotaxis, leukocyte migration, leukocyte chemotaxis, and humoral immune response. In the MF domain, their influence was predominantly associated with specialized functions, including RAGE receptor binding, Toll-like receptor binding, and long-chain fatty acid binding. Furthermore, when considering the CC category, these genes demonstrated noteworthy enrichment in vital compartments such as the secretory granule lumen, cytoplasmic vesicle lumen, and vesicle lumen, underscoring their significance within these intracellular domains. Moreover, our meticulous exploration through KEGG analysis revealed compelling evidence of the enrichment of these genes within the Osteoclast differentiation pathway.

### 3.3. Identification of DFU‐related biomarkers

**Figure 4.**
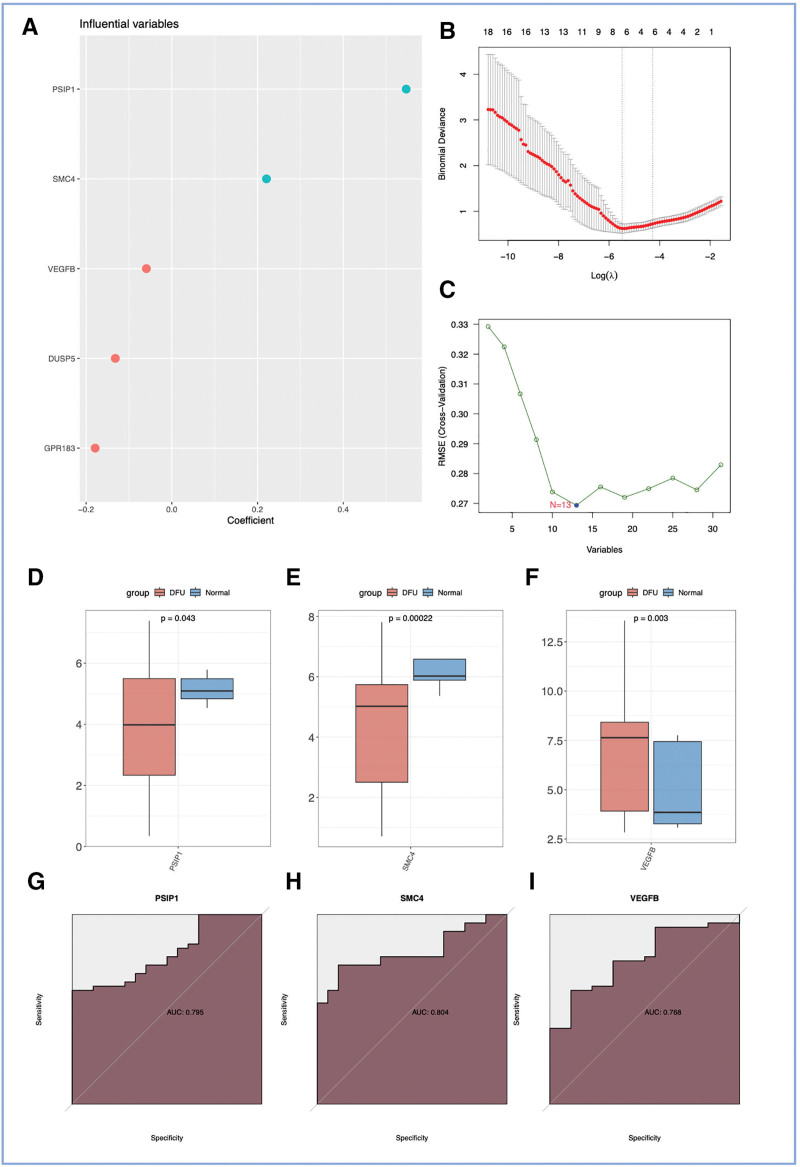
Machine learning-based identification and validation of hub gene VEGFB as a potential biomarker for DFU. (A–C) Feature selection plots from 3 machine learning algorithms applied to the 31 NKT cell-related DEGs. (A) Variable trajectory plot from the Elastic Net regression model. Each curve represents a gene, with the x-axis showing the L1 norm and the y-axis showing the coefficient value. The vertical dashed line indicates the lambda value selected by 10-fold cross-validation. (B) Lasso coefficient profile plot. The vertical dashed line indicates the optimal lambda (lambda.min) where the minimal cross-validation error was achieved. (C) Variable importance plot from the support vector machine (SVM) algorithm, showing the importance score of each gene. (D–F) Validation of candidate gene expression. (D, E) Box plots showing the mRNA expression levels of *PSIP1* (D) and *SMC4* (E) in normal (control) and DFU samples. ***Indicates *P* < .001. (F) Box plot showing the mRNA expression level of VEGFB in normal and DFU samples. ***Indicates *P* < .001. (G–I) Receiver operating characteristic (ROC) curves evaluating the diagnostic performance of the candidate genes. (G, H) ROC curves for *PSIP1* (G, AUC = 0.795) and *SMC4* (H, AUC = 0.804). (I) ROC curve for VEGFB (AUC = 0.768), demonstrating its diagnostic potential for DFU. DEGs = differentially expressed genes, DFU = diabetic foot ulcer, NKT = natural killer T, mRNA = messenger RNA, SVM = support vector machine, *VEGFB* = *vascular endothelial growth factor B*.

To discern potential biomarkers intricately linked to DFU, we employed a comprehensive ensemble of 3 advanced machine learning methodologies: Elasticnet, Lasso, and SVM. Through the Elasticnet method, we identified 5 different genes that exhibited specific associations with DFU among NKT cell-related genes (Fig. 4A). The Lasso algorithm revealed an additional set of 7 feature genes (Fig. 4B). Additionally, the SVM algorithm identified 13 distinct genes (Fig. 4C). Finally, by combining the unique genes identified by the 3 algorithms, we identified *VEGFB*, *PSIP1*, and *SMC4*.

Analysis of *VEGFB*, *PSIP1*, and *SMC4* expressions in normal and DFU samples revealed significant differences. In normal samples, *PSIP1* and *SMC4* exhibited higher expression levels compared to DFU samples (Fig. 4D and E). Conversely, *VEGFB* expression was significantly elevated in DFU samples compared to control samples (Fig. 4F). ROC analysis was performed to assess the clinical diagnostic accuracy of *VEGFB*, *PSIP1*, and *SMC4*. The AUC values for *PSIP1* and *SMC4* were 0.795 and 0.804, respectively (Fig. 4G and H). *VEGFB* also exhibited a promising AUC value of 0.768 (Fig. 4I), further supporting its clinical significance in DFU diagnosis. Based on these findings, *VEGFB* was selected for inclusion in subsequent analyses due to its unique expression pattern and diagnostic potential.

The ROC analysis yielded an AUC value of 0.768 for *VEGFB* (Fig. 4I), indicating a statistically significant but moderate diagnostic accuracy for distinguishing DFU from normal samples. While this suggests *VEGFB* has potential as a biomarker, its standalone diagnostic power may be limited. The moderate AUC could be attributed to the pathophysiological heterogeneity of DFU patients or the influence of other confounding factors not accounted for in this model. Future studies validating *VEGFB* in combination with other clinical parameters or biomarkers may improve diagnostic performance.

### 3.4. GSEA analysis of DFU-related biomarkers

**Figure 5.**
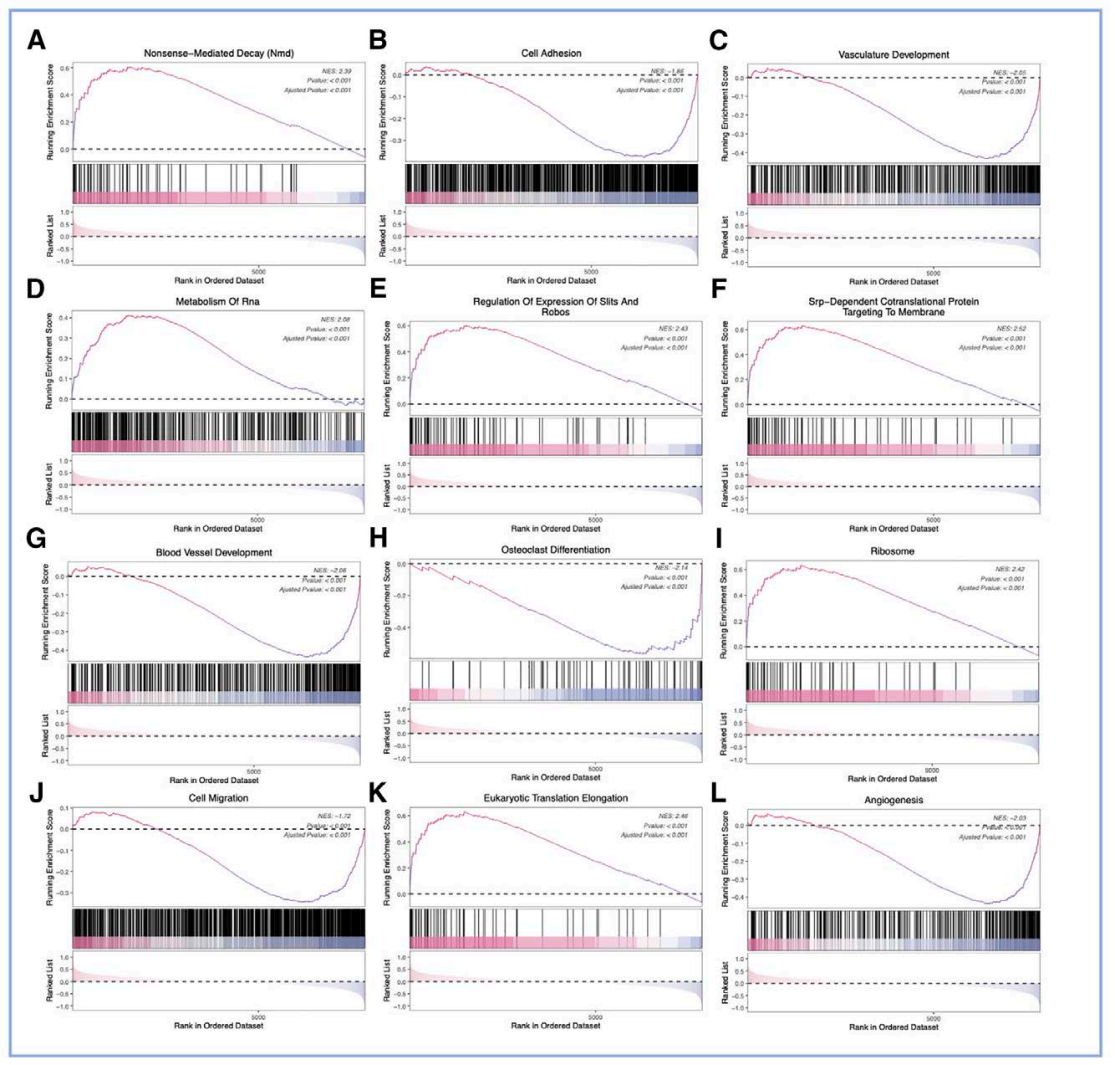
Gene set enrichment analysis (GSEA) reveals biological pathways associated with VEGFB expression in DFU. (A–L) Select GSEA enrichment plots for gene sets significantly associated with high VEGFB expression. Each plot shows the running enrichment score (green line) for the gene set as the analysis moves down the ranked list of genes (based on correlation with VEGFB). The leading-edge subset of genes that primarily contribute to the enrichment signal is highlighted as vertical black lines on the x-axis. The plots display enrichment for pathways including (but not limited to): (A) cell adhesion molecules, (B) vasculature development, (C) RNA metabolism, and (D) angiogenesis. DFU = diabetic foot ulcer, RNA = ribonucleic acid, *VEGFB* = *vascular endothelial growth factor B*.

We conducted GSEA on the DFU-related biomarkers to illustrate *VEGFB*’s significance in DFU (Fig. 5A–L). The results revealed that *VEGFB* participates in multiple biological processes, including cell adhesion, vasculature development, metabolism of ribonucleic acid, cell migration, and angiogenesis signaling pathway. This suggests that *VEGFB* may play a significant role in the etiology of DFU and provides direction for further research.

### 3.5. Identification of DFU-related biomarkers‐interacting genes

**Figure 6.**
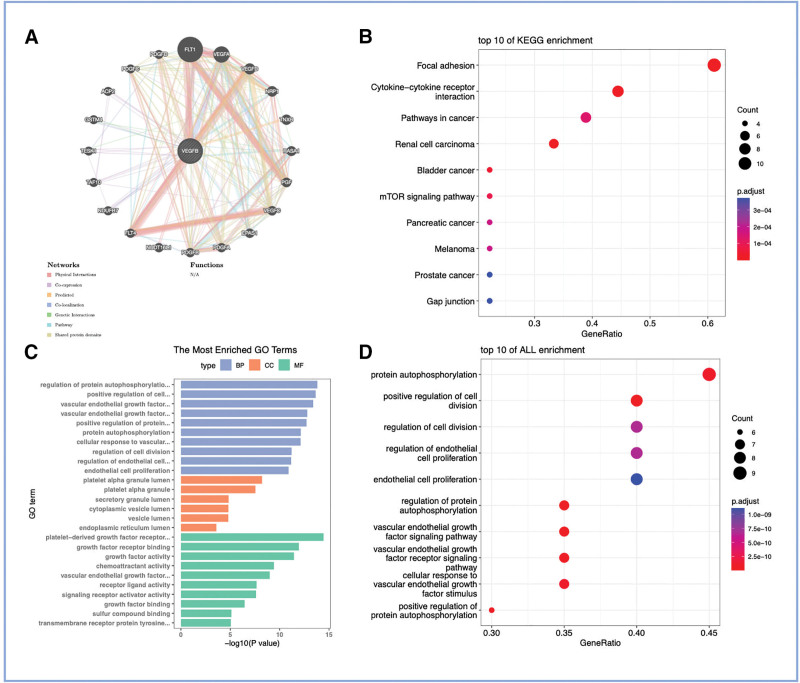
Protein-protein interaction network and functional analysis of VEGFB and its associated genes. (A) Interaction network of VEGFB and its top 20 predicted functional partners, generated using GeneMANIA. The network includes physical interactions, co-expression, pathway sharing, and predicted interactions. The node color represents the query gene (VEGFB, center) and the associated genes. (B–D) Functional enrichment analysis of the genes in the interaction network. (B) Bar plot of the top significantly enriched KEGG pathways. (C) Bar plot of the top significantly enriched gene ontology (GO) biological process terms. (D) Bar plot of the top significantly enriched GO molecular function terms. KEGG = Kyoto Encyclopedia of Genes and Genomes, *VEGFB* = *vascular endothelial growth factor B*.

Using GeneMANIA, we created an interactome for *VEGFB* and its associated genes. A total of 20 genes were found to be linked to *VEGFB* (Fig. 6A). Furthermore, in KEGG analysis, these genes were notably linked to various biological processes, including focal adhesion, cytokine–cytokine receptor interaction, and pathways in cancer. In GO analysis, these genes were associated with various biological processes, including regulation of protein autophosphorylation, positive regulation of cell.

### 3.6. Correlation between DFU-related biomarkers and immune cells

**Figure 7.**
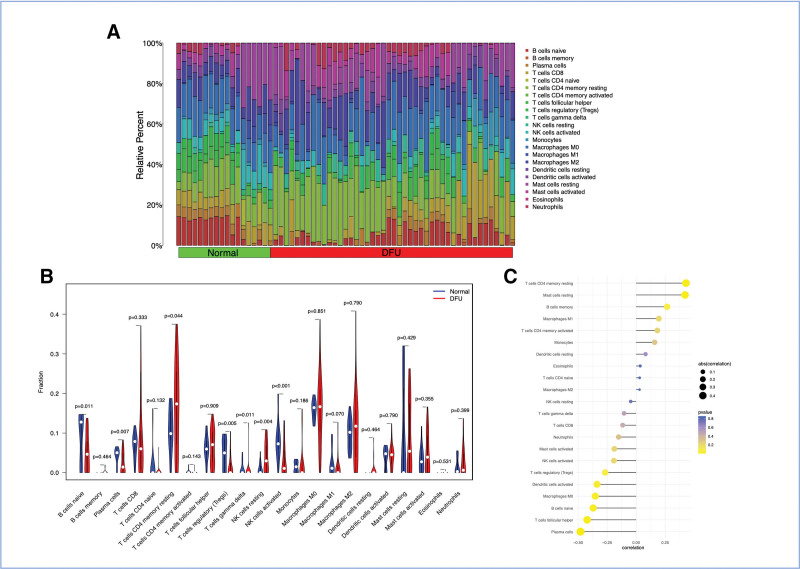
Immune infiltration landscape and its correlation with VEGFB expression in DFU. (A) Bar plot showing the relative proportions of 22 immune cell types in each sample from the DFU cohort, as estimated by CIBERSORT. (B) Box plots comparing the infiltration levels of significantly different immune cell types between normal and DFU samples. *P < .05, *P* < .01, *P < .001 (Wilcoxon test). Cell types with a prefix of “N” indicate normal samples, and “D” indicate DFU samples. (C) Correlation heatmap between VEGFB expression and the infiltration levels of various immune cells. The color scale represents the Pearson correlation coefficient (*r*), with red indicating positive correlation and blue indicating negative correlation. *Indicates a statistically significant correlation (*P* < .05). DFU = diabetic foot ulcer, *VEGFB* = *vascular endothelial growth factor B*.

Additionally, using the CIBERSORT method, we analyzed an analysis on each sample within the DFU cohort to ascertain of immune cells (Fig. 7A). In comparison to normal samples, the findings revealed a notable elevation in the concentrations of plasma cells, T cells CD4 memory resting, Tregs, and NK cell resting in DFU samples. In contrast, levels of B cells naive, NK cell active were substantially increased in normal samples (Fig. 7B). *VEGFB* showed associations with multiple immune cells, including T cells CD4 memory resting, mast cells resting B cells memory (Fig. 7C). On the other hand, *VEGFB* demonstrated strong negative correlations with plasma cells, T cells follicular helper, B cells naïve, macrophages M0, dendritic cells activated, Tregs (Fig. 7C).

The correlation analysis revealed that *VEGFB* expression levels were significantly associated with the infiltration levels of multiple immune cells (Fig. 7C). For instance, the positive correlation with resting CD4 + memory T cells and negative correlation with Tregs and naive B cells suggest a potential role for *VEGFB* in modulating the adaptive immune landscape within the DFU microenvironment. We hypothesize that *VEGFB*, beyond its known metabolic and vascular functions, may influence the recruitment or functional polarization of these immune cells through paracrine signaling. Specifically, its negative correlation with Tregs, which are known to suppress inflammation and potentially impair wound healing in chronic ulcers, might indicate a mechanism by which *VEGFB* contributes to a pro-inflammatory state that hinders resolution. These intriguing correlations warrant further experimental validation to elucidate the causal relationships and underlying mechanisms.

## 4. Discussion

DFU is a severe complication of DM, affecting approximately 1/3 of diabetic patients during their lifetime.^[12]^ With a global prevalence of 18.6 million and 1.6 million cases in the United States annually, DFU poses a significant burden on healthcare systems.^[7]^ Approximately half of DFU cases develop infections, leading to partial or total foot amputation in 20% of cases.^[7]^ Hence, an immediate imperative exists to identify specific therapeutic objectives for enhancing the outlook for patients with DFU. NKT cells, characterized by their expression of both T-cell receptor and NK cell receptor, have garnered significant interest due to their capacity for cytokine secretion and cytotoxicity reminiscent of NK cells.^[13]^ Studies have shown reduced expression of NK cell activating receptors and impaired NK cell activity in patients with long-term diabetes.^[14,15]^ Nonetheless, the involvement of NKT cells in DFU remains uncertain. To address this gap, this study employed single-cell transcriptome analysis to identify specific NKT cell subsets associated with DFU. Furthermore, machine learning was integrated to explore the abnormal expression of NKT cell-related genes in DFU, which was subsequently validated through experimentation. These findings enhance comprehension regarding the involvement of NKT cells in DFU and offer potential insights for developing innovative therapeutic approaches.

In systemic circulation, *VEGFB*, as a constituent of the *VEGF* family, stands out for its lesser contribution to the development of new blood vessels compared to *VEGF-A*.^[16]^ Its main function is in the formation of new blood vessels. In a mouse model of diabetes, *VEGFB* may inhibit or potentially reverse the advancement of type 2 diabetes.^[11]^ This is achieved by enhancing vascular density in adipose tissue, thereby reducing inflammation and improving insulin function in obese mice. Recent studies indicate that *VEGFB* regulates angiogenesis by suppressing the FGF2/FGFR1 pathway,^[17]^ potentially linking this mechanism to its involvement in the pathological process of diabetes. Furthermore, RL-QN15 has been found to promote DFU wound healing via the p38 mitogen-activated protein kinase and Smad3/miR-4482-3p/*VEGFB* axis.^[18]^ *VEGFB* is also the target of various drugs,^[19]^ including aminoglutethimide (used in the treatment of multiple myeloma), pegaptanib sodium (used for diabetic macular edema), sorafenib (a first-line treatment for liver cancer), bevacizumab, and BaiXiPu (targeted drugs for metastatic colorectal cancer and other tumors). The results suggest that *VEGFB* holds promise as a novel therapeutic target for DFU. However, these studies did not elucidate the source of *VEGFB* expression and its interaction with specific immune cell populations. The novelty of this study is that, by integrating single-cell sequencing and bulk transcriptome data, we localized the first-time upregulation of *VEGFB* specifically to NKT cells in DFU lesions. This finding, validated by multiple machine learning algorithms, suggests a novel pathological mechanism: NKT cell-derived *VEGFB* may act as a critical bridge connecting immune abnormalities to vascular dysfunction in DFU. Therefore, our study not only confirms the presence of *VEGFB* in DFU but, more importantly, elucidates a novel cellular source (NKT cells) and a novel potential mode of action (immune-vascular crosstalk), which provides a new perspective for understanding the complex microenvironment of DFU.

Based on its significant differential expression, association with the immune microenvironment, and existing drug targeting properties, our data suggest that *VEGFB* is worthy of further exploration as a potential therapeutic target for DFU. We speculate that targeting *VEGFB* signaling in NKT cells may provide a novel strategy to regulate the immune microenvironment of DFU wound healing. However, this speculation requires subsequent functional experiments (e.g., using agonist/antagonist or conditional gene knockout techniques in animal models) to verify its causal relationship and therapeutic potential.

Despite the valuable insights presented in this study through bioinformatics analysis, several limitations remain. First, our findings are based primarily on retrospective analyses of data from public databases. Despite the use of a batch-correction algorithm, potential residual batch effects and heterogeneity across data sets may still have an effect on the results. Second, the sample size of this study, especially the relatively small sample size of the single-cell dataset, may have limited our ability to detect more subtle changes. Most importantly, the results of the present study are correlational, and there is a lack of direct in vitro and in vivo functional experiments to verify the causal role of *VEGFB* on DFU progression in NKT cells. Future studies should focus on validating VEGFB expression in larger prospective clinical cohorts and using cell coculture and gene-edited mouse models to functionally explore the molecular mechanism of NKT cell-derived VEGFB in DFU wound healing. Such work will be a critical step in translating current computational findings into tangible clinical value.

## 5. Conclusion

Ultimately, this research provides insights into the mechanism of action of NKT cells in DFU and clarifies the underlying pathways. We identified *VEGFB* as a potential therapeutic target for DFU. The results contribute to our understanding of DFU pathogenesis and suggest new avenues for the development of targeted therapies.

## Author contributions

**Conceptualization:** Jianming Chen, Weilun Wang, Yao He.

**Methodology:** Tao You.

**Investigation:** Meihong Lei.

**Writing – original draft:** Jianming Chen, Weilun Wang, Yao He, Tao You, Meihong Lei.

**Writing – review & editing:** Jianming Chen, Weilun Wang.
